# Application of Machine Learning in Predicting Hepatic Metastasis or Primary Site in Gastroenteropancreatic Neuroendocrine Tumors

**DOI:** 10.3390/curroncol30100668

**Published:** 2023-10-19

**Authors:** Mahesh Kumar Padwal, Sandip Basu, Bhakti Basu

**Affiliations:** 1 Molecular Biology Division, Bhabha Atomic Research Centre, Mumbai 400085, India; mkpadwal@barc.gov.in; 2 Homi Bhabha National Institute, Mumbai 400094, India; drsanb@yahoo.com; 3 Radiation Medicine Centre, Bhabha Atomic Research Centre, Tata Memorial Hospital Annexe, Mumbai 400012, India

**Keywords:** machine learning, gene features, RNA-SEQ, neuroendocrine tumors, hepatic metastasis, primary site, random forest

## Abstract

Gastroenteropancreatic neuroendocrine tumors (GEP-NETs) account for 80% of gastroenteropancreatic neuroendocrine neoplasms (GEP-NENs). GEP-NETs are well-differentiated tumors, highly heterogeneous in biology and origin, and are often diagnosed at the metastatic stage. Diagnosis is commonly through clinical symptoms, histopathology, and PET-CT imaging, while molecular markers for metastasis and the primary site are unknown. Here, we report the identification of multi-gene signatures for hepatic metastasis and primary sites through analyses on RNA-SEQ datasets of pancreatic and small intestinal NETs tissue samples. Relevant gene features, identified from the normalized RNA-SEQ data using the mRMRe algorithm, were used to develop seven Machine Learning models (LDA, RF, CART, k-NN, SVM, XGBOOST, GBM). Two multi-gene random forest (RF) models classified primary and metastatic samples with 100% accuracy in training and test cohorts and >90% accuracy in an independent validation cohort. Similarly, three multi-gene RF models identified the pancreas or small intestine as the primary site with 100% accuracy in training and test cohorts, and >95% accuracy in an independent cohort. Multi-label models for concurrent prediction of hepatic metastasis and primary site returned >98.42% and >87.42% accuracies on training and test cohorts, respectively. A robust molecular signature to predict liver metastasis or the primary site for GEP-NETs is reported for the first time and could complement the clinical management of GEP-NETs.

## 1. Introduction

Neuroendocrine tumors constitute a well-differentiated group within the highly heterogeneous tumors originating from the diffuse neuroendocrine system, comprised of cells with neuronal and endocrine functions [1]. GEP-NETs account for approximately 2% of all gastrointestinal tumors, with pancreatic NETs (pNETs) and small intestinal NETs (siNETs) representing about 80% of GEP-NETs [2]. Recent Surveillance, Epidemiology, and End Results (SEER) data have indicated a rising incidence of GEP-NETs at 6.98 per 100,000 [3]. Often diagnosed at the metastatic stage due to their indolent and slow-growing nature, GEP-NETs present vague symptoms resembling common abdominal conditions, thus contributing to diagnostic delays [4]. Determinants for treatment regimens and overall survival include the primary site, tumor grade, and metastasis. Presently, immunohistological markers such as Chromogranin A, synaptophysin (for neuroendocrine origin), lineage-specific transcription factors (e.g., TTF-1, ISL, PDX-1 for the primary site), and cytokeratin (for epithelial origin) are used [5]. However, primary sites remain unconfirmed in approximately 20% of cases termed as CUP-NETs (Cancer of Unknown Primary–Neuroendocrine Tumors) [6]. Tissue-based molecular diagnostics for metastasis detection or primary site confirmation remain unexplored.

Leveraging RNA-SEQ-generated large-scale gene expression data has proven effective in identifying biomarkers for cancer detection, metastasis prediction, cancer sub-type classification, and prognosis [7,8]. For instance, microRNA panels have facilitated reliable classification and grading of GEP-NETs [9]. Integrated RNA and DNA sequencing was useful to establish the drivers of metastatic breast cancer [10]. Machine learning algorithms are a popular choice in cancer research as they aid in sifting through extensive datasets to identify pertinent genes while eliminating unrelated technical variations [11,12]. They have been reliably applied for the classification of tumors [10], identification of carcinogenesis-related genes [13], determination of the primary sites [14], early cancer diagnosis [15], and identification of prognostic genes [16]. However, gene signatures for diagnosing or predicting the primary site or metastasis for GEP-NETs remain enigmatic. The aim of this study was to utilize gene expression data of the primary and metastatic NET tissues [7,17] to delineate gene signatures to accurately predict liver metastasis and primary sites of GEP-NETs.

## 2. Material and Methods

### 2.1. RNA-SEQ Datasets and Processing

RNA-SEQ datasets of NET tissues (*n* = 214) used in this study are listed in Table 1. Raw SRA files were converted to fastq files [https://github.com/rvalieris/parallel-fastq-dump, accessed on 5 April 2022], and read quality was checked with FASTQC [18]. Low-quality reads were removed with Trimmomatic in a single-end mode (parameters: LEADING: 10, TRAILING: 10, SLIDING WINDOW: 4:15, MINILEN: 35) [19]. Filtered reads were aligned to the human genome (hg38 p. 12, Ensemble GTF (Version 101) using a splice-aware alignment tool STAR (Version 2.7.2) [20]. Gene counting was carried out using the STAR.

The raw counts were processed using the DaMiRseq pipeline, described earlier [21]. In brief, raw counts were imported into the Rstudio (version 2022.04.0-7) for the initial filtering. The genes with noisy low expression (mean raw counts <10 in more than 10% of samples) were filtered out. The datasets were normalized using variance stabilization transformation (VST), as described previously [22], and samples with a Spearman correlation coefficient <0.7 were filtered out. Surrogate variable analysis (SVA) was carried out and expression data were adjusted for surrogate variables (SVs) unrelated to the hepatic metastasis or primary site of tumors.

### 2.2. Feature Selection, Model Building, and Performance Evaluation

RNA-SEQ dataset (GSM2626909, *n* = 182), comprising primary (*n* = 126) and liver metastasis (*n* = 56) samples, was randomly split into the training (70%) and test (30%) sets using the Caret package (version 6.0.90) [23], to ensure sufficient sample size for the training of the hepatic metastasis model. Feature selection was performed on the training dataset, using the minimum redundancy maximum relevance (mRMRe) package in R [24]. We independently executed mRMRe multiple times (*n* = 500) with 20 features as a target in each execution of feature selection, using default parameters. The gene features selected in all the executions (*n* = 500) were considered for the hepatic metastasis model. To enhance accuracy, we generated feature combination sets (*n* = 503) by varying the number of features from a minimum of 2 to a maximum of 9. A similar approach was employed to identify important features to discriminate between the primary sites of NETs, using only liver metastasis samples of the pNETs (*n* = 30) and siNETs (*n* = 26).

### 2.3. Machine Learning Models and Performance Evaluation 

Seven machine learning algorithms, viz., Linear Discriminant Analysis [LDA], Random Forest [RF], Classification and Regression Tree [CART], Support Vector Machine [SVM], k-Nearest Neighbor [k-NN], Extreme Gradient Boosting [XGB], and Gradient Boosting Machine [GBM] were selected based on their utility for gene expression-based classifications [25,26]. All analyses were carried out in R using the CARET package [23]. We used the “repeated-cv” method with 6-fold and 100 repeats with grid search parameters and ROC as an accuracy metric to train the model. For XGB and GBM models, TuneGrid was used for model parameter optimization. Further accuracy, sensitivity, and specificity were calculated using the confusion matrix command of the caret package [23]. After training the model, its performance was evaluated on the test dataset. A similar approach was used to identify important features to discriminate between the primary sites of the samples. Only liver metastasis samples of the pNETs and siNETs were used for the primary site prediction. For simultaneous prediction of the hepatic metastasis and the primary site, multi-label RF models were built in the Python package sci-kit learn [27]. For multi-label classification, mRMRe-derived gene features (liver metastasis = 9 features; primary site = 12 features) and all the samples (*n* = 182) were used for training, testing, and validation purposes. 

### 2.4. Differential Expression Analyses

Differential expression analysis for the primary versus liver metastasis samples was carried out using the DESeq2 package [28].

### 2.5. Statistical Software and Figures

R statistical programming language (v4.0.2) was used for all calculations and statistical analysis. All the graphs were generated with the R package ggplot2 [29].

### 2.6. Weighted Gene Expression Network Analysis (WGCNA) Construction

Tutorial RScript provided with the R package WGCNA (Ver 1.7.0) [30] was used for step-wise WGCNA network construction. First, the outlier samples were checked and soft power threshold analyses were carried out to find the soft power threshold. The adjacency matrix was calculated using all 24,123 genes and 182 samples, with network type as *signed* network and bicor selected as network cor functions. The TOM matrix was calculated from the adjacency matrix and converted into the dissimilarity matrix by subtracting the TOM matrix from 1 [31]. Dissimilarity TOM matrix was then used for hierarchical clustering and module detection. Branches were cut at the threshold of 0.75 and a minimum of 30 genes for each module. Module–trait relationship, module membership, and gene significance were calculated as described in the tutorial script. Finally, gene-features associated scores for module membership, gene significance, and module–trait correlation were extracted for further analyses.

## 3. Results

### 3.1. Alignment of RNA-SEQ Profiles with the Human Genome, Gene Quantification and Count Normalization

**Figure 1:**
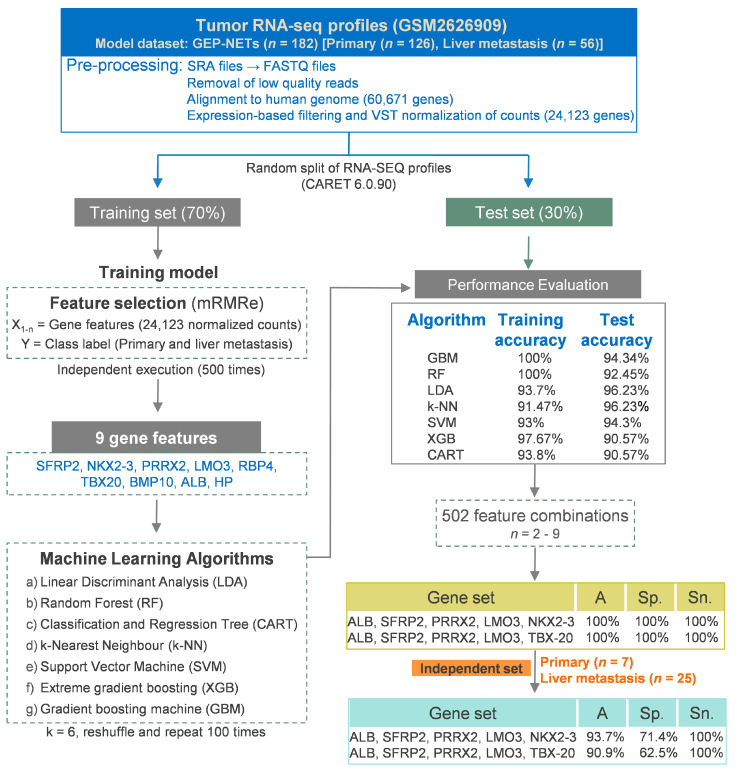
Flowchart of the step-wise procedure describing data acquisition, pre-processing of RNA-SEQ datasets, selection of the important features, building of machine learning models, and performance evaluation for classification of primary versus liver metastasis samples.

The RNA-SEQ datasets (*n* = 182) comprised of primary tumors (pNETs, *n* = 83 and siNETs, *n* = 43) and liver metastases (pNETs, *n* = 30 and siNETs, *n* = 26) samples had an average of 32 million reads. RNA-SEQ datasets were processed as described in Figure 1. After removing low-quality reads, around 90% of all the reads aligned to the human genome (hg38 p. 12, Ensemble GTF (Version 101), and all the samples had 60,671 gene features.

After expression-based filtering, 24,123 gene features were retained for subsequent analyses. All samples exhibited a minimum correlation of 0.7 (Supplementary Figure S1A), following VST normalization of the gene counts. Batch effects related to the sequencing data were removed by SVA, which identified 14 surrogate variables (Supplementary Figure S1B). Three SVs, namely 1, 9, and 12, displayed significant correlations with known biological variables, specifically the sample classes (primary tumors and liver metastases) and primary sites (pNETs and siNETs) (Supplementary Figure S1C). The gene counts were adjusted for the remaining 11 surrogate variables with no significant correlations with either the sample classes or the primary sites.

### 3.2. Hepatic Metastasis Model

#### 3.2.1. Identification of Gene Features Relevant to Hepatic Metastasis

The RNA-SEQ dataset (*n* = 182) was randomly divided into training (70%) and test (30%) sets based on the tumor type (primary versus liver metastasis). The mRMRe algorithm identified nine gene features important for hepatic metastasis classification (Table 2 and Supplementary Table S1) from the training set samples’ VST-normalized and SV-adjusted counts (24,123 genes).

#### 3.2.2. Development of Machine Learning Models and Importance of the Identified Features 

**Figure 2:**
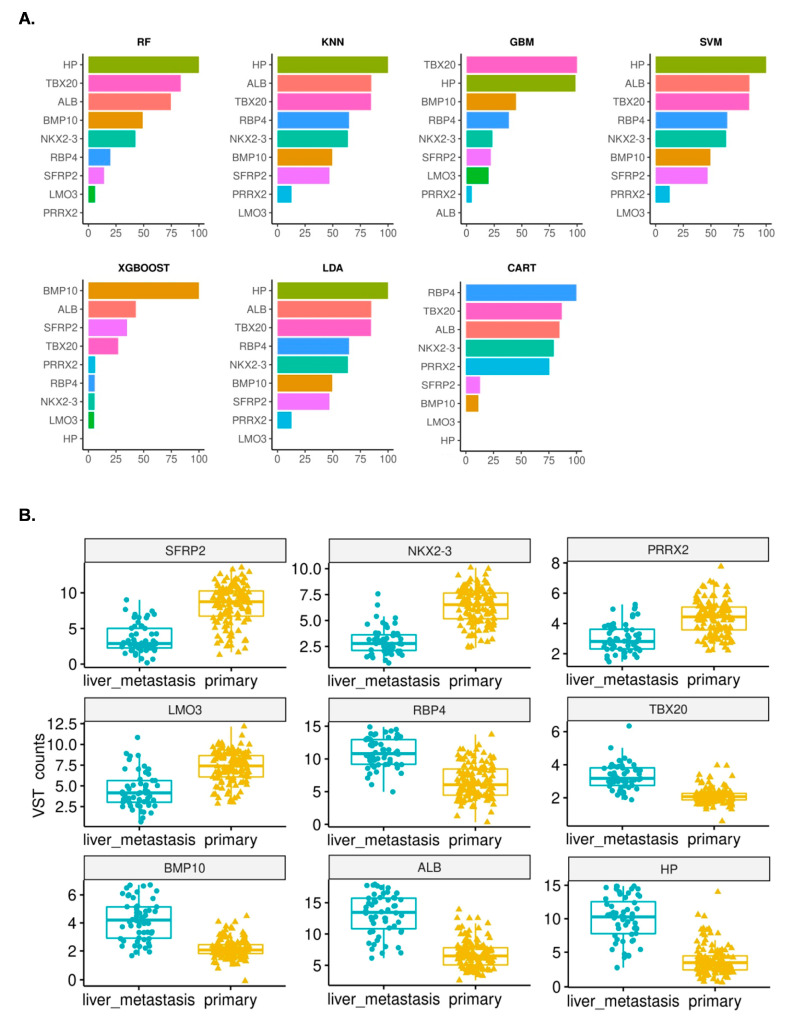
(**A**) Feature importance plots for the gene relevant to hepatic metastasis of NETs. X axis represents relative feature importance. For abbreviations of the models, please see Figure 1. (**B**) Box plots show expression levels of the 9 genes, in the primary and liver metastasis samples. The middle horizontal line represents the median of the VST counts for each sample.

Next, the efficacy of the nine gene features for distinguishing primary tumors from hepatic metastases was assessed with seven distinct ML algorithms: LDA, SVM, CART, RF, k-NN, XGB, and GBM. Across both training and test sets, all seven models achieved >90% accuracy (Figure 1). In the test set, LDA and k-NN attained the highest accuracy of 96.23% (Figure 1). Feature importance analysis revealed Haptoglobin as the key feature in RF, k-NN, SVM, and LDA (Figure 2A). For GBM, XGB, and CART models, TBX20, BMP10, and RBP4, respectively, played pivotal roles in achieving classification accuracy (Figure 2A). Albumin [ALB] was also recognized as the second most important feature in k-NN, SVM, XGB, and LDA (Figure 2A). Importantly, all nine genes demonstrated differential expression between primary tumor and liver metastasis samples (Figure 2B; Table 2).

#### 3.2.3. Concise Gene Signatures Improve Classification Accuracy of the Hepatic Metastasis Model 

Given the distinct significance of each gene feature on individual model prediction accuracy (Figure 2A), we explored whether a reduced number of features and unique feature combinations could enhance classification accuracy. We generated a comprehensive set of 502 distinct feature combinations, encompassing 2 to 9 gene features in each combination (Supplementary Table S2). We evaluated all the feature combination sets for classification accuracy using seven ML algorithms [RF, LDA, k-NN, GBM, SVM, GBM, XGB]. Sensitivity, specificity, and accuracy metrics for all 502 gene feature sets are detailed in Supplementary Table S3. Intriguingly, two RF models, each composed of a 5-gene feature set, demonstrated the highest classification accuracy. Specifically, both models achieved 100% accuracy, sensitivity, and specificity in differentiating primary and metastatic samples, both in the training and test datasets (Table 3 and Supplementary Table S3).

Further, two aforementioned RF models were tested on an independent dataset (GSE118014), encompassing primary (*n* = 25) and liver metastasis (*n* = 7) samples (Table 1). Both RF models displayed accuracy exceeding 90% in accurately classifying the samples (Table 4). Notably, both models achieved higher predicted accuracy compared to the No Information Rate (NIR) (*p*-value < 0.0182).

### 3.3. Primary Site Model

#### 3.3.1. Identification of Gene Features Relevant to the Primary Site

**Figure 3:**
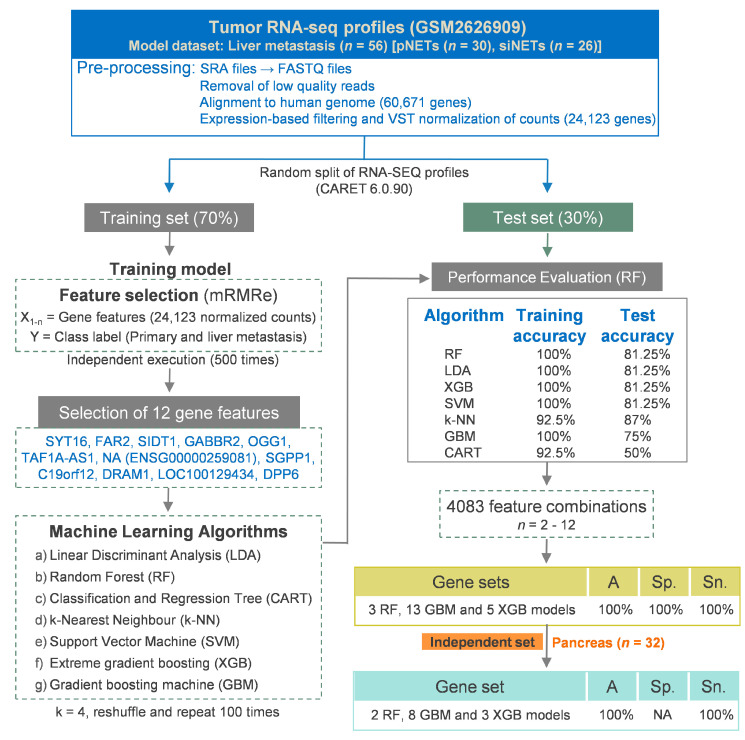
Flowchart of the step-wise procedure describing data acquisition, pre-processing of RNA-SEQ datasets, selection of the important features, building of machine learning models, and performance evaluation for identification of primary site from liver metastasis samples.

Precise determination of the primary site holds significant clinical implications for management and prognosis. To identify gene features specifically relevant to the primary site (pancreas or small intestine), we focused our approach on utilizing liver metastasis samples. This strategy emulates cancers of unknown primary (CUP), aiming to uncover gene features pertinent to the primary tumor site by examining metastatic sites. Liver metastasis samples from pNETs (*n* = 30) and siNETs (*n* = 26) from GSM2626909 were randomly split into training (70%) and test (30%) sets using the Caret package (Figure 3). A set of 12 genes was consistently selected across all 500 feature selection rounds of the mRMRe algorithm (Table 5 and Supplementary Table S4).

#### 3.3.2. Development of Machine Learning Models and Importance of the Identified Features

**Figure 4:**
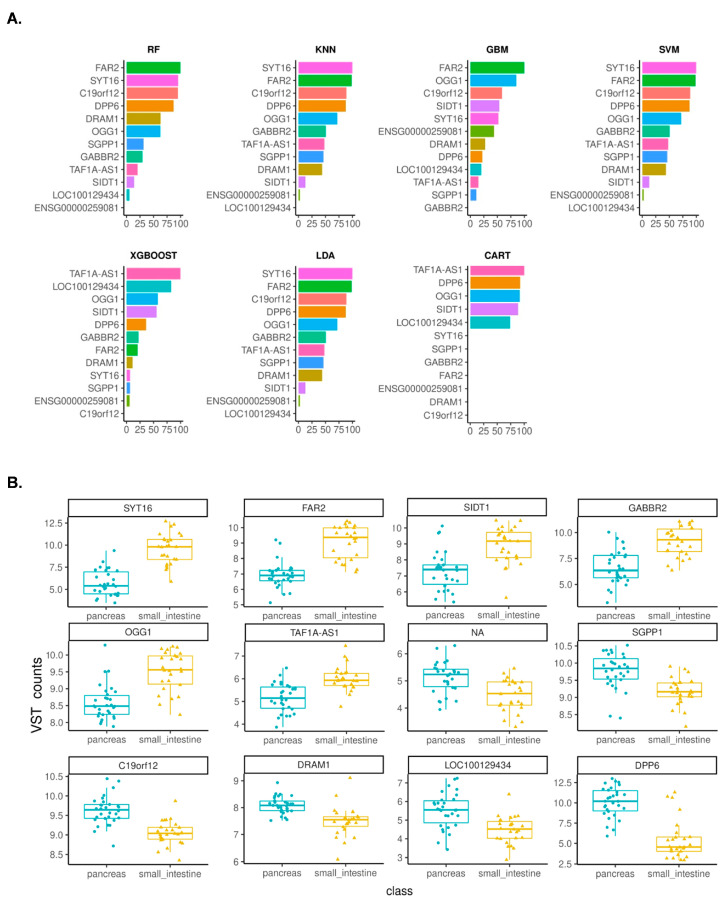
(**A**) Feature importance plots for the gene features associated with the site of primary derived from the liver metastasis samples. X axis represents relative feature importance. For abbreviations of the models, please see Figure 1. (**B**) Box plots showing the expression levels of the 12 gene features in pancreas and small intestine samples. The middle line shows the median values of variant-stabilization transformed counts for each sample. NA represents gene feature ENSG00000259081.

As detailed in the earlier section, ML models were developed using 12 mRMRe-identified gene features, and the classification efficiency of all seven models was evaluated. RF, LDA, XGB, and SVM models returned a similar performance with 100% accuracy in the training set and 81.25% accuracy in the test set (Figure 3). SYT16 gene was identified as the most important feature in KNN, SVM and LDA models, and the second most important feature in the RF model (Figure 4A). FAR2 was identified as the most important feature in RF and GBM models, and as a second most important feature in k-NN and SVM models (Figure 4A). All 12 genes were differentially expressed between the primary tumor and the liver metastasis samples (Figure 4B; Table 5).

#### 3.3.3. Concise Gene Signatures Improve the Classification Accuracy of the Primary Site Model

Similar to the approach used for the hepatic metastasis model, gene combination sets with features ranging from 2 to 12 genes (resulting in 4083 unique combinations) were generated (Supplementary Table S5). We trained all seven ML algorithms using these 4083 feature sets with the training dataset and assessed the model performance using the test dataset (Supplementary Table S6). Among the seven ML algorithms, specifically, three Random Forest (RF) models, fourteen Gradient Boosting Machine (GBM) models, and five Extreme Gradient Boosting (XGB) models were generated using the 21 unique feature sets, and they could classify both the training and test datasets with 100% accuracy (Table 6). Of note, all 12 gene features identified by the mRMRe algorithm are represented in different models. The models’ performance was evaluated on an independent dataset of pNETs, consisting of 32 cases (25 primary and 7 liver metastasis). In this evaluation, two RF models, eight GBM models, and three XGB models achieved 100% classification accuracy in predicting the primary site (Table 7).

### 3.4. A Multi-Label Model to Predict the Primary Site and Hepatic Metastasis of NETs

We further determined whether a single model could predict hepatic metastasis and the primary site. We constructed a single multi-label ensemble Random Forest model using the 21 mRMRe-derived features, consisting of 9 hepatic metastasis prediction gene features and 12 primary site prediction gene features. This model achieved 100% accuracy with the training dataset, comparable to the individual prediction models for hepatic metastasis or primary site. However, the multi-label model outperformed the individual models with the test dataset. Specifically, the multi-label model achieved 96.36% accuracy in predicting hepatic metastases, whereas the individual RF model achieved 92.45% accuracy (Figure 1). Similarly, the multi-label model achieved 90.9% accuracy for primary site prediction, surpassing the 81.25% accuracy of the individual RF model (Figure 3). Thus, the performance of the multi-label model, with all 21 gene features, was superior to that of the individual prediction models for hepatic metastasis or the primary site. Furthermore, we investigated whether concise feature sets could enhance the prediction accuracy of the multi-label model. We generated 42 sets of feature combinations, comprising 2 feature sets (Table 2) and 21 feature sets (Table 5), to train 42 multi-label RF models (Supplementary Table S7). Among these models, 24 achieved 100% accuracy in the training dataset for hepatic metastasis classification. In the test dataset, the highest accuracy of 94.55% was observed for 3 models (Multi-Label 13, 16, and 36) for hepatic metastasis classification (Supplementary Table S7). For predicting the primary site, 24 models achieved 100% accuracy in the training set. In contrast, in the test set 3 models (Multi-Label 15, 18, and 31) achieved 90.91% accuracy (Supplementary Table S7). We also validated the performance of the multi-label models on an independent dataset (Supplementary Table S7). For the prediction of hepatic metastasis, the accuracy ranged from 84.38% to 100% (Supplementary Table S7). However, for the prediction of the primary site, the accuracy ranged from 21.88% to 100% (Supplementary Table S7). It is important to note that no single multi-label model achieved 100% accuracy for both hepatic metastasis and primary site predictions in the training, test, and independent datasets. In total, 14 multi-label models achieved 100% accuracy in the training datasets for predicting hepatic metastasis and the primary site (Table 8). Among these, the top-performing 2 models (Multi-Label 16 and 36) consistently achieved >89.09% accuracy in all the test and independent datasets for both hepatic metastasis and primary site predictions (Table 8).

### 3.5. Weighted Gene Correlation Network Analysis

**Figure 5:**
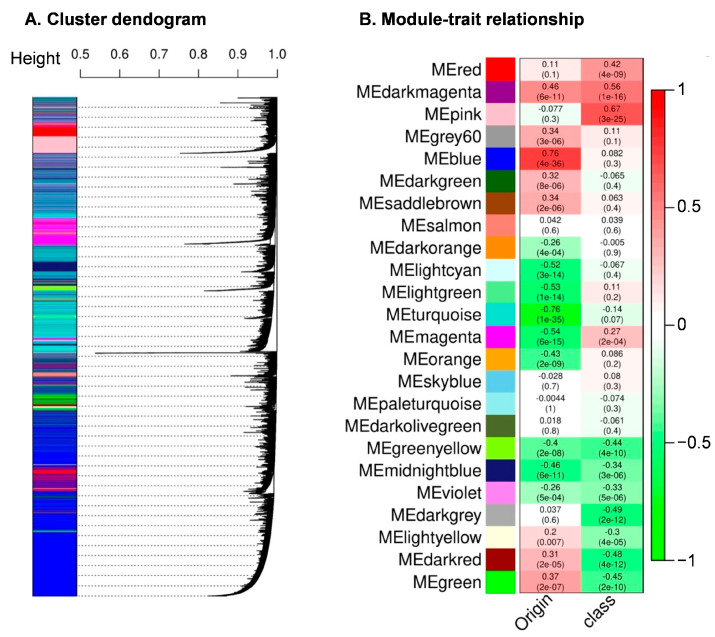
WGCNA network construction and module identification. (**A**) Hierarchical cluster dendrogram showing the identified modules and color assignment of each module. For hierarchical clustering a dissimilarity measure (1-TOM) was used. Highly interconnected groups of genes are shown as branches. Modules are represented in the vertical bar as different colors. In total, 24 modules with the 24,123 genes were detected with WGCNA. (**B**) Heatmap of correlation between modules, class, and site of the origin. (Each cell represents the correlation and its associated *p*-value in a bracket).

Weighted gene correlation network analysis (WGCNA) is a widely used method for identifying important modules containing highly correlated genes associated with the clinical attributes of interest [32,33,34]. We employed WGCNA to investigate the correlation of the mRMRe-identified gene features, which were utilized in our machine learning models, with two key aspects of Neuroendocrine Tumors (NETs): tumor class (primary versus liver metastasis) and primary site (pNETs versus siNETs). The WGCNA network was constructed with VST-normalized counts of genes (*n* = 24,123) from 182 samples (GSM2626909). Following standard WGCNA network construction guidelines, we initially screened for outlier samples through hierarchical clustering (Supplementary Figure S2A). Subsequently, we determined a soft threshold power of 12 (Supplementary Figure S2B) to calculate the adjacency matrix. We aimed to create a scale-free topology network with a minimum module size of 30, utilizing bicor as the correlation type and forming a signed network. The resulting WGCNA network consisted of 24 modules (Figure 5A).

To identify modules significantly associated with the tumor class (primary versus liver metastasis) and primary site (pNETs versus siNETs), we conducted module–trait relationship analyses. These analyses involved assessing correlations between the module eigengenes and the tumor class or site of origin. Notably, we identified a total of seven modules that exhibited significant associations: three positively correlated modules (pink, magenta, and red) and four negatively correlated modules (dark grey, dark red, green, and green-yellow) with the tumor class (correlation coefficient |0.4| or higher and *p*-value < 1 × 10^−9^) (Figure 5B). Crucially, all six gene features that effectively discriminated liver-metastasized NETs from primary NETs were part of these four modules. Additionally, we computed the Gene Module Membership (GMM) for each gene within these modules by assessing its correlation with the eigengene. All six gene features displayed high GMM scores (>0.5). Furthermore, we calculated the Gene Significance Score (GSE) for each gene within these modules by correlating gene expression with the tumor class. All six gene features exhibited high and statistically significant GSE scores (>0.5 and *p*-value > 1 × 10^−9^).

In the context of the primary site (pNETs versus siNETs), we identified nine modules (midnight blue, green-yellow, orange, magenta, turquoise, light green, light cyan, blue, and dark magenta) that were significantly associated with the primary site (correlation coefficient |0.4| or higher and *p*-value < 2 × 10^−8^) (Figure 5B). Two of these modules were positively correlated, while seven were negatively correlated with the primary site. Notably, the six gene features that accurately classified the primary site were found within four of these modules (Table 9).

Through gene significance and module membership analyses, we determined that three of these gene features were part of the blue module and exhibited high module membership scores (Score > 0.33). Additionally, all six features displayed a significant association with the primary site (GSE > 0.25 and *p*-value > 1.38 × 10^−4^) (Table 10). 

## 4. Discussion

In general, metastasis is a dynamic process of dissemination of tumor cells to a target site. This complex process involves molecular reprogramming, the tumor microenvironment, and interactions favoring the target site [35,36]. The liver is a common metastatic site for pancreatic and gastrointestinal tract tumors [37,38]. Gene expression profiles play a crucial role in determining the invasive potential of primary tumor cells [35]. Our study identified nine gene features that accurately differentiated between the primary and the liver metastasis samples. Five genes encode secretory proteins (ALB, HP, BMP10, RBP4, and SFRP2), while four genes encode transcription factors (TBX20, NKX2-3, LMO3, and PRRX2). In primary tumors, SFRP2, NKX2-3, PRRX2, and LMO3 exhibited higher expression levels, while ALB, HP, BMP10, RBP4, and TBX20 showed lower expression compared to liver metastasis samples (Figure 2B).

The genes exhibiting higher expression in primary tumors are functionally linked to metastatic progression. For instance, SFRP2 plays a role in the Wnt signaling pathway, and its expression is regulated by methylation, influencing cell differentiation and growth [39]. SFRP2 is a potential prognostic and diagnostic biomarker in breast and prostate cancers [40,41]. NKX2-3 is down-regulated in the liver metastasis samples of the NETs [42], which corroborates our findings (Figure 2B). NKX2-3 is a homeodomain transcription factor, and it regulates the expression of the M cadherin in endothelial cells and, thus, the migration of leukocytes in tissue [43]. PRRX2 acts as an important transcription factor that regulates miRNA expression related to pulmonary large cell neuroendocrine tumors [44]. The pancreatic NENs with enhanced PDX1 expression were reportedly enriched with PRRX2 [45]. Thus, higher PRRX2 expression in primary tumors (Figure 2B) is in line with the published literature. Over-expression of PRRX2 induces epithelial to mesenchymal transition (EMT) in breast carcinoma [46] and enhances migration and invasiveness in breast cancer [47]. In the primary NETs, up-regulation of PRRX2 may promote EMT. Higher expression of PRRX2 in primary samples may help tumor metastasis since inhibition of PRRX2 has been shown to suppress liver metastasis [48]. A Lim-domain-containing transcription factor LMO3 inhibits the activity of the p53 tumor suppressor [49]. Interaction of LMO3 with another transcription factor, HEN2, is correlated with poor prognosis of neuroblastoma and tumor growth [50]. Epigenetic regulation has been discovered to play a significant role in regulating several crucial genes related to metastasis and overall survival in NETs [51]. Therefore, we investigated the possible regulation of the nine genes related to the hepatic metastasis model and found that SFRP2, NKX2-3, PRRX2, and LMO3 are regulated by epigenetic modifications in several cancer types [52,53,54,55]. Although there is no direct report of the regulation of these genes through epigenetic mechanisms in neuroendocrine tumors, we cannot rule out the possibility of such epigenetic regulation. Expression of ALB and Haptoglobin (HP) is enriched in the liver. ALB has been identified as one of the liver metastasis-associated hub genes in colorectal carcinoma [56]. Similarly, higher levels of blood HP are associated with advanced cancers, distant metastasis, and poor outcomes [57], while higher expression of RBP4 has been correlated with higher metastatic potential, increased invasiveness, and clonogenic potential [58]. We observed lower expression of BMP10 in primary tumors. Low levels of BMP10 were found to be associated with bigger tumor size, worse TNM stage, earlier recurrence, and poorer survival in hepatocellular carcinoma [59]. TBX20 is an important transcription factor involved in heart development and angiogenesis [60]. Lichtenauer et al. have shown that TBX20 acts via the PROK2-PRKR2 pathway in the angiogenesis in colorectal cancer [61].

Three genes, SFRP2, NKX2-3, and LMO3, identified in this study as important features associated with liver metastasis of NETs, belong to the metastatic gene signature derived for pNETs [62]. Similarly, SFRP2, NKX2-3, and ALB were also a part of the machine learning model for the prediction of liver metastasis in colorectal adenocarcinoma [63]. Thus, these genes may represent a common signature for liver metastases irrespective of the microenvironments at the primary or the metastatic site. Taken together, hepatic metastasis-associated gene features reported in this study are potential markers for metastatic NETs and are worth pursuing for clinical applications. 

Primary sites cannot be confirmed in about 20% of NET cases [6]. Such CUP-NET patients cannot benefit from the therapies designed for specific tissue types. Thus, accurate determination of the site of primary is an important task. Gene expression signatures serve as valuable markers for tracing the primary tumor site. This study identified 12 gene features that accurately differentiated between pNETs and siNETs from liver metastasis samples. The expression of SYT16, FAR2, SIDT1, GABBR2, OGG1, and TAF1A-AS1 was higher in siNETs, while the expression of DPP6, DRAM1, SGPP1, LOC100129434, NA (ENSG00000259081), and C19orf12 was higher in pNETs (Figure 4B). 

SYT16 is a calcium-independent synaptogamin involved in membrane trafficking [64]. SYT16 has higher expression in the siNETs than the pNETs (Figure 4B). FAR2, SIDT1, and OGG1 show higher expression levels in the gastrointestinal tract than in the pancreas (https://www.proteinatlas.org/, accessed on 22 December 2022). GABBR2 is a part of the GABA_b_ receptor signaling. GABA_b_ receptors are expressed throughout the small intestine and are involved in the secretion of the inhibitory neuron. In pancreatic Beta cells, the expression of GABBR2 is regulated while GABBR1 is constitutively expressed [65]. This may be why GABBR2 expression is higher in the liver-metastasized siNET samples.

Conversely, DPP6 has approximately 25-fold higher expression in the pancreatic alpha and beta cells than in the proximal tissue [66]. SGPP1 is a phosphatase involved in sphingolipid metabolism and regulates calcium signaling [67]. Genes that code for antisense RNA (TAF1A-AS1), lncRNA (ENSG00000259081), and ORF (C19orf12) remain uncharacterized. Given the contrasting expression patterns of DPP6 and SYT16, we explored whether the ratio of VST counts of DPP6 to SYT16 could facilitate the straightforward discrimination of pNETs from siNETs. A DPP6/SYT16 ratio greater than one was observed in 100% of pNET samples, while the ratio was less than one in 81% of siNET samples (Supplementary Table S8). The ratio DPP6/SYT16 can be useful in distinguishing the pNETs and siNETs from the liver metastasis samples. We propose that our approach can seamlessly integrate with existing clinical processes if transcript expression can be confirmed alongside immunohistochemistry (IHC) and pathology assessments for neuroendocrine origin confirmation. This integration not only has the potential to reduce additional costs but also enhances overall feasibility, making it a practical and valuable option for clinical applications. Further, these transcript expressions can also be estimated in blood profiles of NET patients for easy determination in pNETs or siNETs origin. This underscores the applicability of our findings in real-world clinical settings, where accurate diagnosis and classification of neuroendocrine tumors are paramount.

## 5. Conclusions

With the availability of high-quality RNA-SEQ datasets of cancer tissues in the public domain, machine learning is poised to dramatically transform diagnosis and therapeutic management, predictably translating into better prognosis. Machine learning algorithms pick up subtle changes in the expression data and are suitable for developing a multi-gene model to distinguish the classifiers confidently. In this communication, we used GEP-NETs as a test system to evaluate the applicability of machine learning to predict either hepatic metastasis or the primary site. We conclude that the gene features extracted from the NET-tissue RNA-SEQ profiles can differentiate the classes under investigation with very high accuracy. Further, our study also clearly demonstrates that concise gene signatures perform better. In the future, this stratagem may complement the clinical management of cancer patients.

## 6. Limitation of the Study

This study did not investigate how conventional pathology including immunohistochemistry performs in comparison with gene expression algorithms in correctly predicting the primary site of metastases.

## Figures and Tables

**Table 1:** RNA-SEQ datasets used in this study.

GEO Accession	pNETs	siNETs	Purpose	Reference
GSE98894	*n* = 113	*n* = 69	Training and Test sets	[7]
GSE118014	*n* = 32	*n* = 0	Independent validation set	[17]

**Table 2:** Hepatic metastasis model: Top gene features identified by mRMRe algorithm.

Sr. No.	Symbol	Description	LOG2FC (Primary/Liver mets)	padj
1.	SFRP2	Secreted frizzled related protein 2	5.51	8.35 × 10^−34^
2.	NKX2-3	NK2 homeobox 3	4.33	1.36 × 10^−33^
3.	PRRX2	Paired related homeobox 2	1.94	1.74 × 10^−7^
4.	LMO3	LIM domain only 3	1.86	2.95 × 10^−5^
5.	RBP4	Retinol binding protein 4	−2.83	4.17 × 10^−6^
6.	TBX20	T-box transcription factor 20	−3.8	2.53 × 10^−15^
7.	BMP10	Bone morphogenetic protein 10	−8.09	2.36 × 10^−43^
8.	ALB	Albumin	−10.06	7.71 × 10^−106^
9.	HP	Haptoglobin	−10.28	4.69 × 10^−87^

**Table 3:** Hepatic metastasis model: Performance evaluation on training and test sets.

Gene Sets	Accuracy	Specificity	Sensitivity
HM-RF1: ALB, SFRP2, PRRX2, LMO3, NKX2-3	100%	100%	100%
HM-RF2: ALB, SFRP2, PRRX2, LMO3, TBX20	100%	100%	100%

**Table 4:** Hepatic metastasis model: Performance validation on independent set.

Models	Accuracy	Sensitivity	Specificity	95% Confidence Interval
HM-RF1	93.75%	71.43%	100%	0.7567–0.9923
HM-RF2	90.91%	62.5%	100%	0.7567–0.9808

**Table 5:** Primary site model: Top gene features identified by mRMRe algorithm.

Sr. No.	Symbol	Description	Log2FC (siNET/pNET)	padj
1.	SYT16	Synaptotagmin 16	3.01	9.88 × 10^−6^
2.	FAR2	Fatty acyl-CoA reductase 2	1.78	1.62 × 10^−8^
3.	SIDT1	SID1 transmembrane family member 1	1.48	1.34 × 10^−5^
4.	GABBR2	Gamma-amino butyric acid type B receptor subunit 2	1.32	0.02
5.	OGG1	8-oxoguanine DNA glycosylase	1.02	2.85 × 10^−9^
6.	TAF1A-AS1	TAF1A antisense RNA 1	0.79	0
7.	ENSG00000259081	lncRNA	−0.59	0.02
8.	SGPP1	Sphingosine-1-phosphate phosphatase 1	−0.67	3.09 × 10^5^
9.	C19orf12	Chromosome 19 open reading frame 12	−0.69	0
10.	DRAM1	DNA damage-regulated autophagy modulator 1	−0.78	2.94 × 10^−6^
11.	LOC100129434	Uncharacterized LOC100129434	−2.01	4.66 × 10^−9^
12.	DPP6	Dipeptidyl peptidase like 6	−3.63	1.29 × 10^−7^

**Table 6:** Primary site model: Performance evaluation on training and test datasets. The models listed below showed 100% accuracy, specificity, and sensitivity.

Sr. No.	Model *: Gene Features
1	PS-RF1: DPP6, GABBR2, SYT16, SGPP1
2	PS-RF2: DPP6, GABBR2, SYT16, SGPP1, TAF1A-AS1
3	PS-RF3: DPP6, GABBR2, SYT16, SGPP1, LOC100129434
4	PS-GBM1: GABBR2, FAR2
5	PS-GBM2: SYT16, SGPP1, C19orf12
6	PS-GBM3: TAF1A-AS1, GABBR2, FAR2, SYT16
7	PS-GBM4: TAF1A-AS1, GABBR2, FAR2, SGPP1
8	PS-GBM5: LOC100129434, GABBR2, SGPP1, C19orf12
9	PS-GBM6: LOC100129434, SYT16, SGPP1, C19orf12
10	PS-GBM7: SIDT1, DPP6, DRAM1, SYT16
11	PS-GBM8: GABBR2, FAR2, SYT16, SGPP1
12	PS-GBM9: GABBR2, SYT16, SGPP1, C19orf12
13	PS-GBM10: TAF1A-AS1, GABBR2, SYT16, SGPP1, C19orf12
14	PS-GBM11: LOC100129434, OCG1, GABBR2, SYT16, SGPP1, C19orf12
15	PS-GBM12: LOC100129434, DPP6, GABBR2, SYT16, SGPP1, C19orf12
16	PS-GBM13: LOC100129434, GABBR2, SYT16, SGPP1, ENSG00000259081, C19orf12
17	PS- XGB1: SIDT1, DPP6, SYT16, SGPP1
18	PS-XGB2: LOC100129434, DPP6, GABBR2, SYT16, ENSG00000259081
19	PS- XGB3: DPP6, DRAM1, SYT16, SGPP1, C19orf12
20	PS- XGB4: DPP6, GABBR2, SYT16, SGPP1, ENSG00000259081, C19orf12
21	PS- XGB5: TAF1A-AS1, SIDT1, DPP6, GABBR2, FAR2, DRAM1, SYT16, SGPP1, ENSG00000259081

* PS = primary site.

**Table 7:** Primary site model: Performance validation on independent datasets.

Sr. No.	Models	Accuracy	Sensitivity	Specificity
1	PS-RF1: DPP6, GABBR2, SYT16, SGPP1	100%	100%	100%
2	PS-RF3: DPP6, GABBR2, SYT16, SGPP1, LOC100129434	100%	100%	100%
3	PS-GBM2: SYT16, SGPP1, C19orf12	100%	100%	100%
4	PS-GBM5: LOC100129434, GABBR2, SGPP1, C19orf12	100%	100%	100%
5	PS-GBM6: LOC100129434, SYT16, SGPP1, C19orf12	100%	100%	100%
6	PS-GBM9: GABBR2, SYT16, SGPP1, C19orf12	100%	100%	100%
7	PS-GBM10: TAF1A-AS1, GABBR2, SYT16, SGPP1, C19orf12	100%	100%	100%
8	PS-GBM11: LOC100129434, OCG1, GABBR2, SYT16, SGPP1, C19orf12	100%	100%	100%
9	PS-GBM12: LOC100129434, DPP6, GABBR2, SYT16, SGPP1, C19orf12	100%	100%	100%
10	PS-GBM13: LOC100129434, GABBR2, SYT16, SGPP1, ENSG00000259081, C19orf12	100%	100%	100%
11	PS- XGB2: LOC100129434, DPP6, GABBR2, SYT16, ENSG00000259081	100%	100%	100%
12	PS- XGB3: DPP6, DRAM1, SYT16, SGPP1, C19orf12	100%	100%	100%
13	PS- XGB4: DPP6, GABBR2, SYT16, SGPP1, ENSG00000259081, C19orf12	100%	100%	100%

**Table 8:** Top-performing multi-label models: Performance evaluation on training and test datasets and performance validation on independent datasets.

Model_Name Accuracy (%)	Training Metastasis	Test Metastasis	Training Origin	Test Origin	Independent Metastasis	Independent Origin
Multi-label-16	100	94.55	100	89.09	93.75	96.88
Multi-label-36	100	94.55	100	89.09	96.88	93.75
Multi-label-27	100	92.73	100	87.27	84.38	34.38
Multi-label-38	100	92.73	100	87.27	90.63	53.13
Multi-label-40	100	92.73	100	87.27	90.63	78.13
Multi-label-29	100	92.73	100	85.45	90.63	62.50
Multi-label-21	100	92.73	100	83.64	93.75	81.25
Multi-label-18	100	90.91	100	90.91	93.75	62.50
Multi-label-31	100	90.91	100	90.91	90.63	75.00
Multi-label-30	100	90.91	100	89.09	90.63	28.13
Multi-label-32	100	90.91	100	89.09	87.50	100
Multi-label-41	100	90.91	100	89.09	87.50	87.50
Multi-label-17	100	90.91	100	87.27	87.50	59.38
Multi-label-28	100	90.91	100	87.27	90.63	53.13

**Table 9:** WGCNA identified gene significance (GSE), gene significance *p*-value (GSP), and gene module membership score (GMM) for the six gene features of hepatic metastasis models.

Ensemble ID	Gene Name	Module	GSE	GSP	GMM
ENSG00000167157	PRRX2	Midnight blue	−0.50	3.67 × 10^−13^	0.66
ENSG00000163631	ALB	Pink	0.75	1.96 × 10^−34^	0.85
ENSG00000164532	TBX20	Pink	0.68	2.43 × 10^−26^	0.70
ENSG00000119919	NKX2-3	Dark red	−0.70	1.24 × 10^−28^	0.71
ENSG00000145423	SFRP2	Midnight blue	−0.66	6.91 × 10^−24^	0.47
ENSG00000048540	LMO3	Green-yellow	−0.54	2.25 × 10^−15^	0.46

**Table 10:** WGCNA identified gene significance (GSE), gene-significance *p*-value (GSP), and gene module membership score (GMM) of the six gene features of the primary site model.

Ensemble ID	Gene Name	Module	GSE	GSP	GMM
ENSG00000136928	GABBR2	Blue	0.43	2.13 × 10^−9^	0.55
ENSG00000139973	SYT16	Blue	0.64	3.40 × 10^−22^	0.74
ENSG00000126821	SGPP1	Light green	−0.45	2.04 × 10^−10^	0.53
ENSG00000225265	TAF1A-AS1	Blue	0.28	1.38 × 10^−4^	0.33
ENSG00000233251	LOC100129434	Midnight blue	−0.29	5.79 × 10^−5^	0.41
ENSG00000130226	DPP6	Turquoise	−0.63	2.18 × 10^−21^	0.78

## Data Availability

RNA-Seq datasets used in this study are available in public databases. Accession numbers are provided in the manuscript at appropriate places.

## Supplementary Materials

The following supporting information can be downloaded at: https://www.mdpi.com/article/10.3390/curroncol30100668/s1, Figure S1: (A) Samples correlation plot showing clustering of samples based on the primary site of the origin and class of the tumor. (B) Surrogate variable analysis showing a number of the significant variables explaining 95% variance in samples. (C) SVA correlation plot showing the correlation of biological variables with 14 surrogate variables. Figure S2: (A) Hierarchical cluster of samples used for construction of WGCNA network. (B) Plot showing soft threshold power cutoff on the scale independent and network mean connectivity. Table S1: List of mRMRe-identified gene features selected in 500 rounds, for finding gene relevant to hepatic metastasis.; Table S2: Feature combination sets, comprising 2 to 9 genes in each set, for hepatic metastasis model. Table S3: Sensitivity, Accuracy and specificity matrix for 7 ML models generated from 502 feature combination sets for hepatic metastasis model. Table S4: List of mRMRe-identified gene features selected in 500 rounds, for finding gene relevant to primary site. Table S5: Feature combination sets, comprising 2 to 12 genes in each set, for primary site model. Table S6: Sensitivity, Accuracy and specificity matrix for 7 ML models generated from 4083 feature combination sets for primary site model. Table S7: Multi-label models for prediction hepatic metastasis and primary site: Performance evaluation on training and test sets, and performance validation on independent set. Table S8: VST normalized counts ratio of the DPP6/SYT16 in the siNETs versus pNETs liver-metastasis samples.
