# Supplementary material for: Application of Machine Learning in Predicting Hepatic Metastasis or Primary Site in Gastroenteropancreatic Neuroendocrine Tumors
Source: Curr Oncol. 2023 Oct 19;30(10):9244–61. doi: 10.3390/curroncol30100668 (PMC10605255; doi:10.3390/curroncol30100668)
Supplement: Supplementary file 1 [file curroncol-30-00668-s001.zip › Padwal et al., - Supplementary Figures.pptx]

## Slide 1
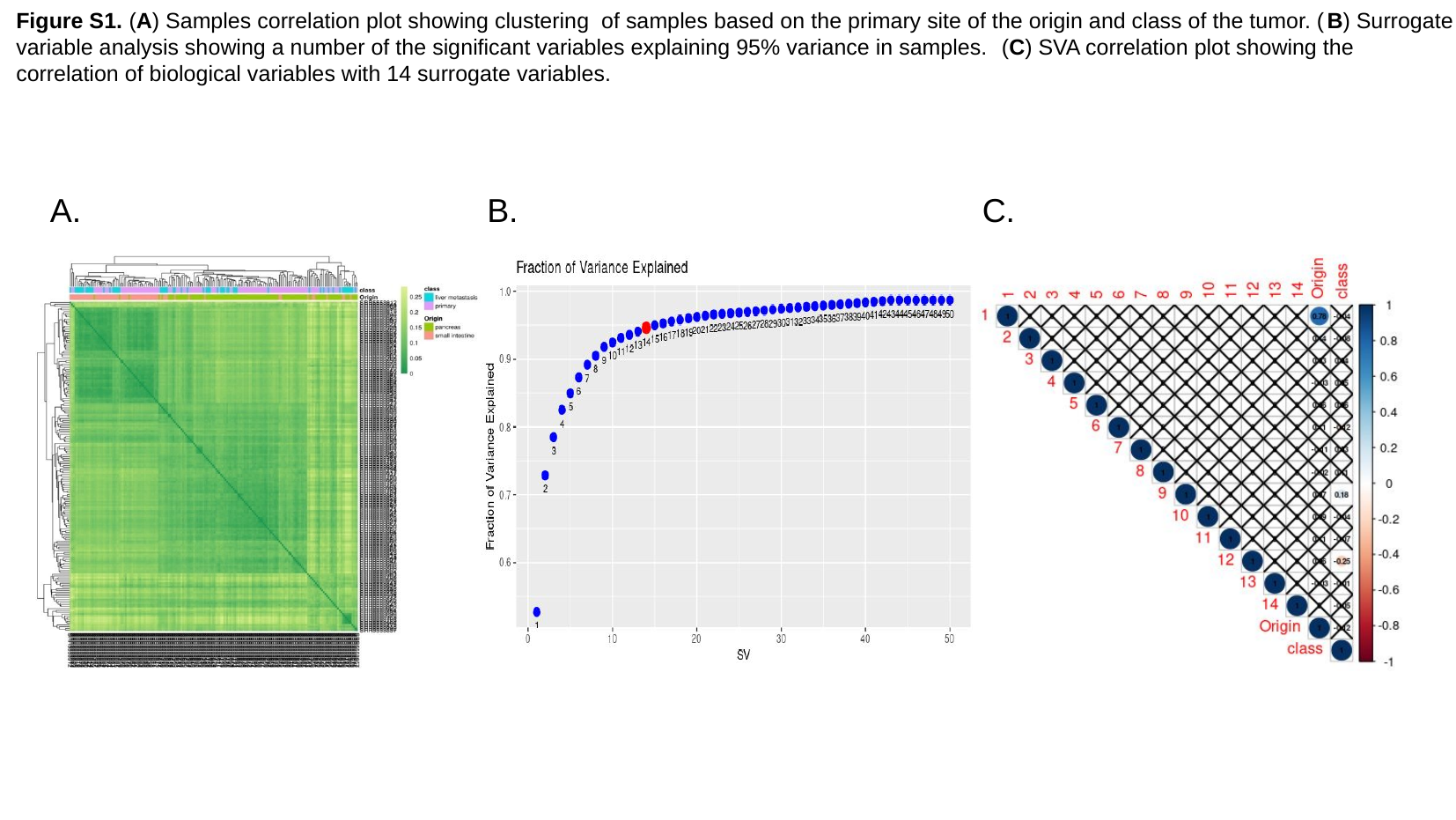

Figure S1. (A) Samples correlation plot showing clustering of samples based on the primary site of the origin and class of the tumor. (B) Surrogate variable analysis showing a number of the significant variables explaining 95% variance in samples. (C) SVA correlation plot showing the correlation of biological variables with 14 surrogate variables.
A.
B.
C.

## Slide 2
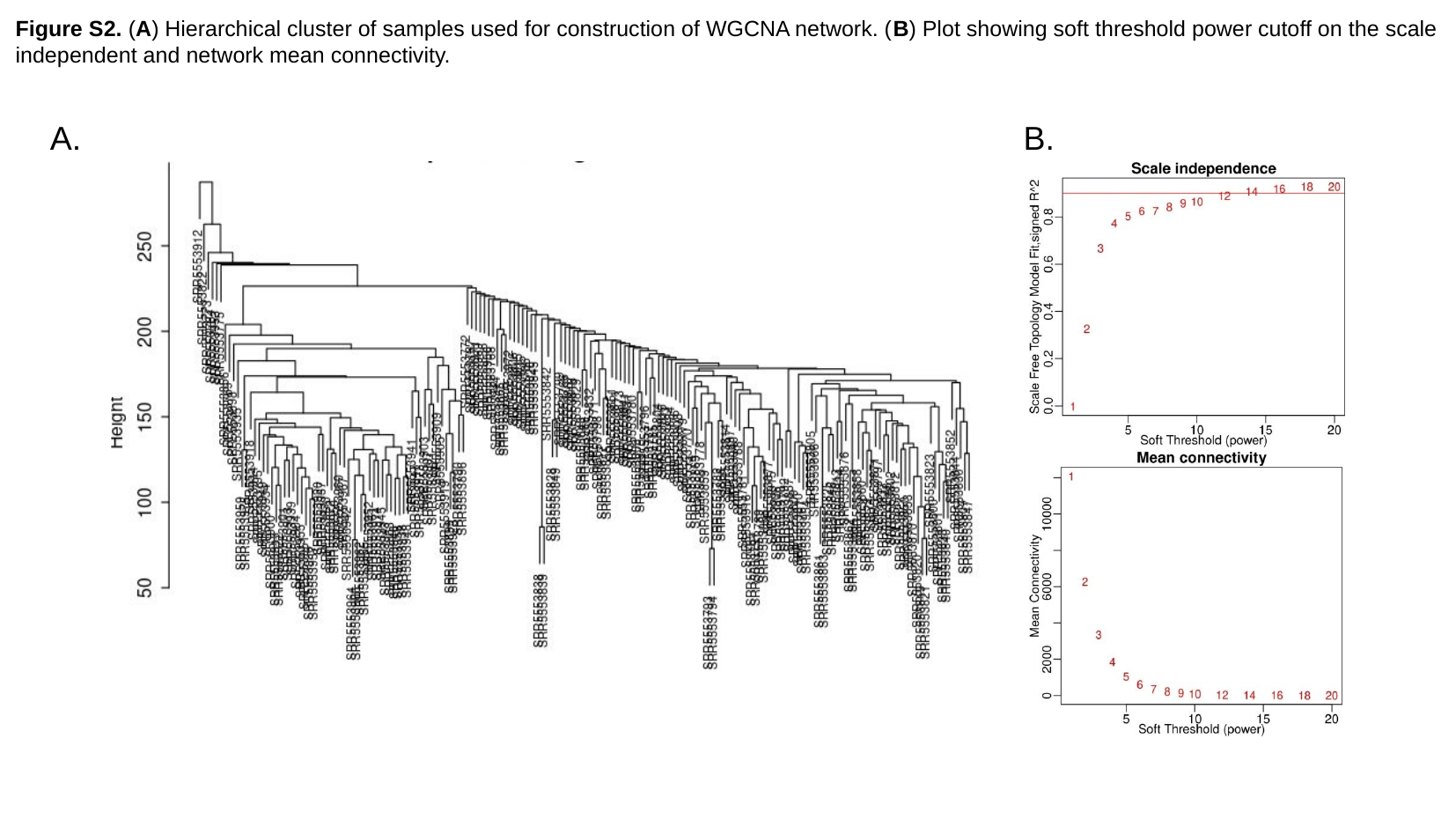

Figure S2. (A) Hierarchical cluster of samples used for construction of WGCNA network. (B) Plot showing soft threshold power cutoff on the scale independent and network mean connectivity.
A.
B.
